# Supplementary material for: Assessing the heterogeneity in the transmission of infectious diseases from time series of epidemiological data
Source: PLoS One. 2023 May 30;18(5):e0286012. doi: 10.1371/journal.pone.0286012 (PMC10228818; doi:10.1371/journal.pone.0286012)
Supplement: S1 Text — Visualization of the data provided in S1 Data. (PDF) [file pone.0286012.s005.pdf]

S1 Text: Survey on statistical distributions of disease intervals

We collected the parameters of statistical distributions of COVID-19 disease intervals reported in literature. The figures in this supplement visualize the respective probability density functions. The sources are also referenced in the main text. In the inference of the reporting offset distributions we use averaged versions of the distributions found in literature. These distributions are also contained in S1 Data and shown in the figures as solid lines.

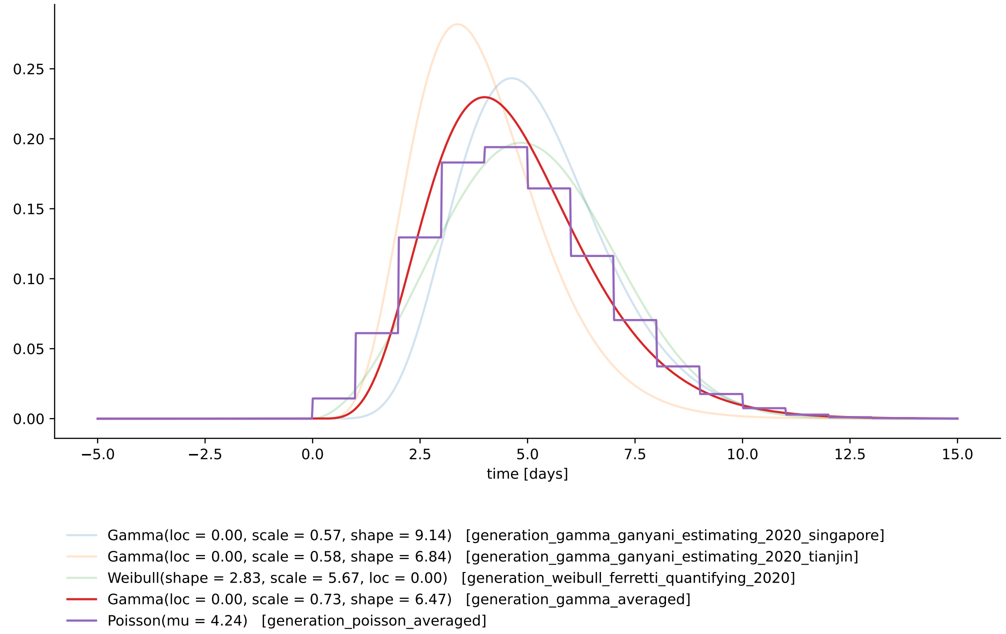

Fig A. Generation interval distributions.

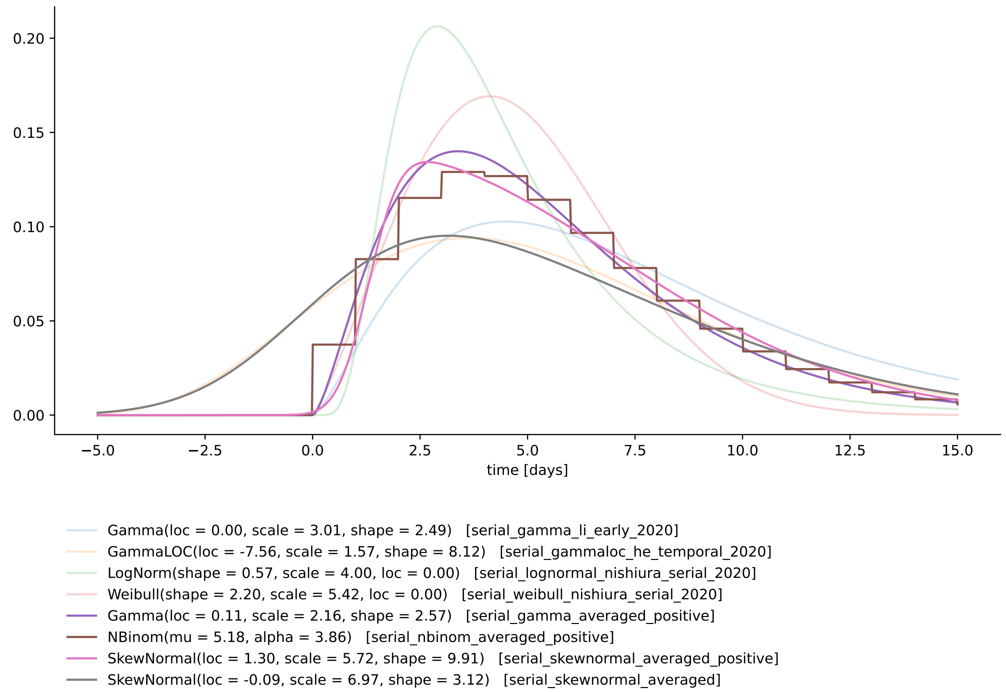

Fig B. Serial interval distributions. We distinguish between models with positive support ('pSI') and models that also allow negative serial intervals ('nSI').

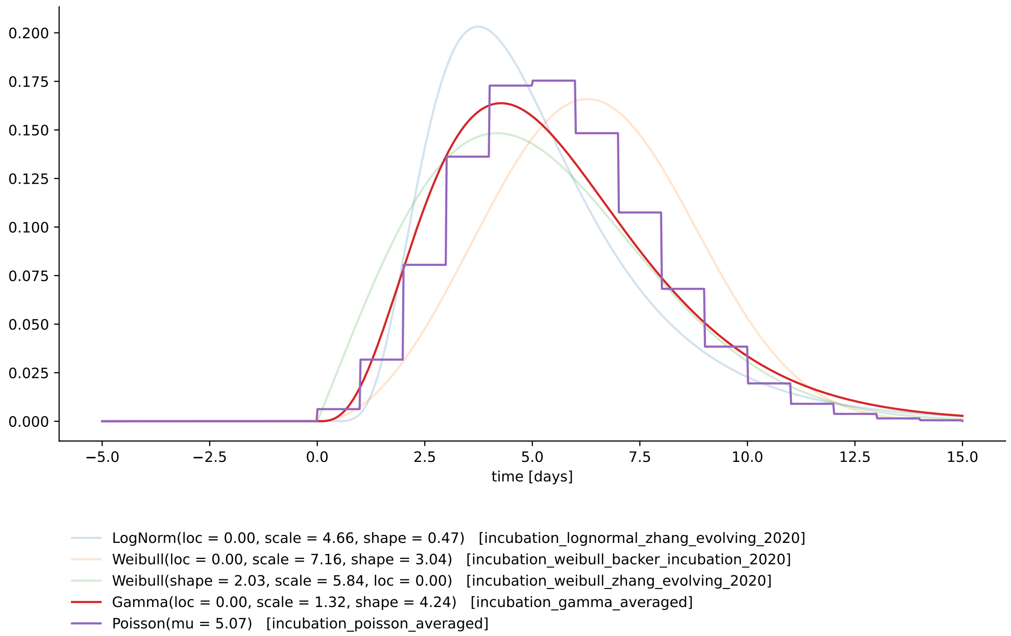

Fig C. Incubation period distributions.

nishiura_serial_2020 Nishiura, H.; Linton, N. M. & Akhmetzhanov, A. R.
Serial interval of novel coronavirus (COVID-19) infections
International Journal of Infectious Diseases, Elsevier BV, 2020, 93, 284-286
doi:10.1016/j.ijid.2020.02.060

ferretti_quantifying_2020 Ferretti, L.; Wymant, C.; Kendall, M.; Zhao, L.; Nurtay, A.; Abeler-Dörner, L.; Parker, M.; Bonsall, D. & Fraser, C.
Quantifying SARS-CoV-2 transmission suggests epidemic control with digital contact tracing
Science, American Association for the Advancement of Science (AAAS), 2020, 368, eabb6936
doi:10.1126/science.abb6936

ali_serial_2020 Ali, S. T.; Wang, L.; Lau, E. H. Y.; Xu, X.-K.; Du, Z.; Wu, Y.; Leung, G. M. & Cowling, B. J.
Serial interval of SARS-CoV-2 was shortened over time by nonpharmaceutical interventions
Science, American Association for the Advancement of Science (AAAS), 2020, 369, 1106-1109
doi:10.1126/science.abc9004

du_serial_2020 Du, Z.; Xu, X.; Wu, Y.; Wang, L.; Cowling, B. J. & Meyers, L. A.
Serial Interval of COVID-19 among Publicly Reported Confirmed Cases
Emerging Infectious Diseases, Centers for Disease Control and Prevention (CDC), 2020, 26, 1341-1343
doi:10.3201/eid2606.200357

li_early_2020 Li, Q.; Guan, X.; Wu, P.; Wang, X.; Zhou, L.; Tong, Y.; Ren, R.; Leung, K. S.; Lau, E. H.; Wong, J. Y.; Xing, X.; Xiang, N.; Wu, Y.; Li, C.; Chen, Q.; Li, D.; Liu, T.; Zhao, J.; Liu, M.; Tu, W.; Chen, C.; Jin, L.; Yang, R.; Wang, Q.; Zhou, S.; Wang, R.; Liu, H.; Luo, Y.; Liu, Y.; Shao, G.; Li, H.; Tao, Z.; Yang, Y.; Deng, Z.; Liu, B.; Ma, Z.; Zhang, Y.; Shi, G.; Lam, T. T.; Wu, J. T.; Gao, G. F.; Cowling, B. J.; Yang, B.; Leung, G. M. & Feng, Z.
Early Transmission Dynamics in Wuhan, China, of Novel Coronavirus–Infected Pneumonia
New England Journal of Medicine, Massachusetts Medical Society, 2020, 382, 1199-1207
doi:10.1056/nejmoa2001316

zhang_evolving_2020 Zhang, J.; Litvinova, M.; Wang, W.; Wang, Y.; Deng, X.; Chen, X.; Li, M.; Zheng, W.; Yi, L.; Chen, X.; Wu, Q.; Liang, Y.; Wang, X.; Yang, J.; Sun, K.; Longini, I. M.; Halloran, M. E.; Wu, P.; Cowling, B. J.; Merler, S.; Viboud, C.; Vespignani, A.; Ajelli, M. & Yu, H.
Evolving epidemiology and transmission dynamics of coronavirus disease 2019 outside Hubei province, China: a descriptive and modelling study
The Lancet Infectious Diseases, Elsevier BV, 2020, 20, 793-802
doi:10.1016/s1473-3099(20)30230-9

lauer_incubation_2020 Lauer, S. A.; Grantz, K. H.; Bi, Q.; Jones, F. K.; Zheng, Q.; Meredith, H. R.; Azman, A. S.; Reich, N. G. & Lessler, J.
The Incubation Period of Coronavirus Disease 2019 (COVID-19) From Publicly Reported Confirmed Cases: Estimation and Application
Annals of Internal Medicine, American College of Physicians, 2020, 172, 577-582
doi:10.7326/m20-0504

backer_incubation_2020 Backer, J. A.; Klinkenberg, D. & Wallinga, J.
Incubation period of 2019 novel coronavirus (2019-nCoV) infections among travellers from Wuhan, China, 20–28 January 2020
Eurosurveillance, European Centre for Disease Control and Prevention (ECDC), 2020, 25
doi:10.2807/1560-7917.es.2020.25.5.2000062

he_temporal_2020 He, X.; Lau, E. H. Y.; Wu, P.; Deng, X.; Wang, J.; Hao, X.; Lau, Y. C.; Wong, J. Y.; Guan, Y.; Tan, X.; Mo, X.; Chen, Y.; Liao, B.; Chen, W.; Hu, F.; Zhang, Q.; Zhong, M.; Wu, Y.; Zhao, L.; Zhang, F.; Cowling, B. J.; Li, F. & Leung, G. M.
Temporal dynamics in viral shedding and transmissibility of COVID-19
Nature Medicine, Springer Science and Business Media LLC, 2020, 26, 672-675
doi:10.1038/s41591-020-0869-5

richter_schaetzung_2020 Richter, L.; Schmid, D.; Chakeri, A.; Maritschnik, S.; Pfeiffer, S. & Stadlober, E.
Schätzung des seriellen Intervalles von COVID19, Österreich
Austrian Agency for Health and Food Safety (AGES), Austrian Agency for Health and Food Safety (AGES), 2020

ganyani_estimating_2020 Ganyani, T.; Kremer, C.; Chen, D.; Torneri, A.; Faes, C.; Wallinga, J. & Hens, N.
Estimating the generation interval for coronavirus disease (COVID-19) based on symptom onset data, March 2020
Eurosurveillance, European Centre for Disease Control and Prevention (ECDC), 2020, 25
doi:10.2807/1560-7917.es.2020.25.17.2000257

ng_estimating_2021 Ng, S. H.-X.; Kaur, P.; Kremer, C.; Tan, W. S.; Tan, A. L.; Hens, N.; Toh, M. P.; Teow, K. L. & Kannapiran, P.
Estimating Transmission Parameters for COVID-19 Clusters by Using Symptom Onset Data, Singapore, January–April 2020
Emerging Infectious Diseases, Centers for Disease Control and Prevention (CDC), 2021, 27, 582-585
doi:10.3201/eid2702.203018

jhu_incubation see lauer_incubation_2020
