# Supplementary material for: Assessing the heterogeneity in the transmission of infectious diseases from time series of epidemiological data
Source: PLoS One. 2023 May 30;18(5):e0286012. doi: 10.1371/journal.pone.0286012 (PMC10228818; doi:10.1371/journal.pone.0286012)
Supplement: S2 Text — Visualization of the statistical distributions provided in S2 Data. (PDF) [file pone.0286012.s006.pdf]

S2 Text: Visualization of delays in case reporting

Visualization of the parameterized reporting delay distributions provided in S2 Data.

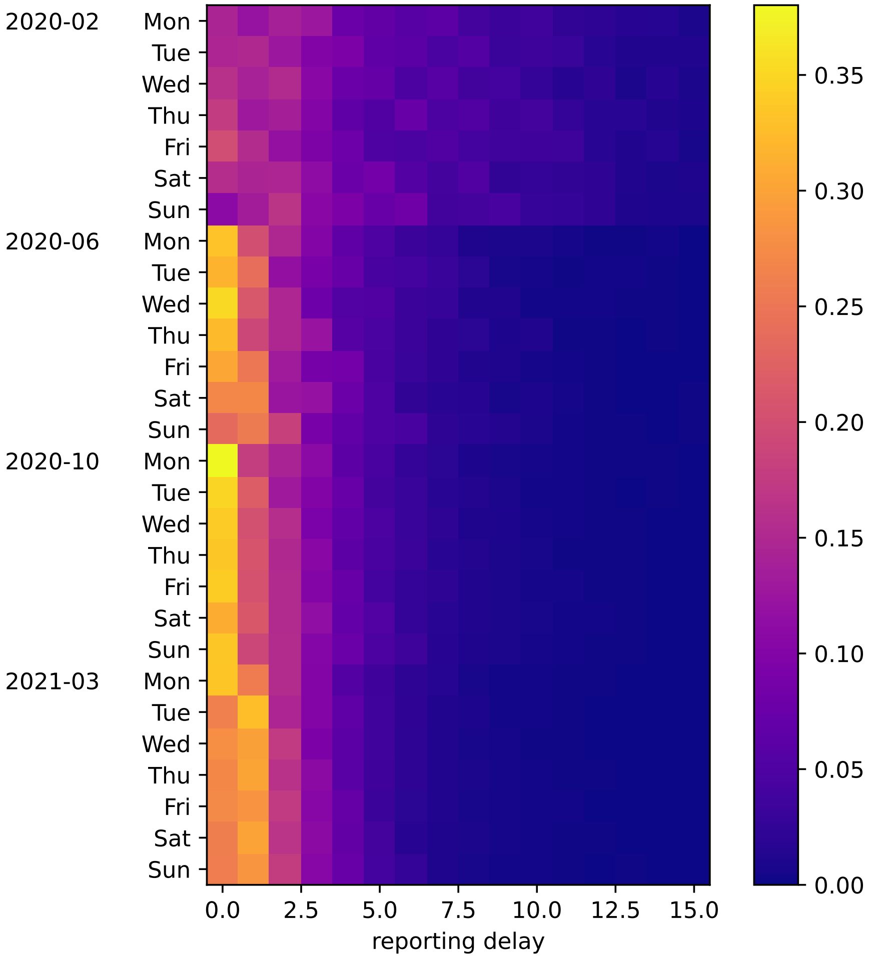

Fig A. Reporting delay distributions displayed separately for four time periods ('phases') and for each day of the week.

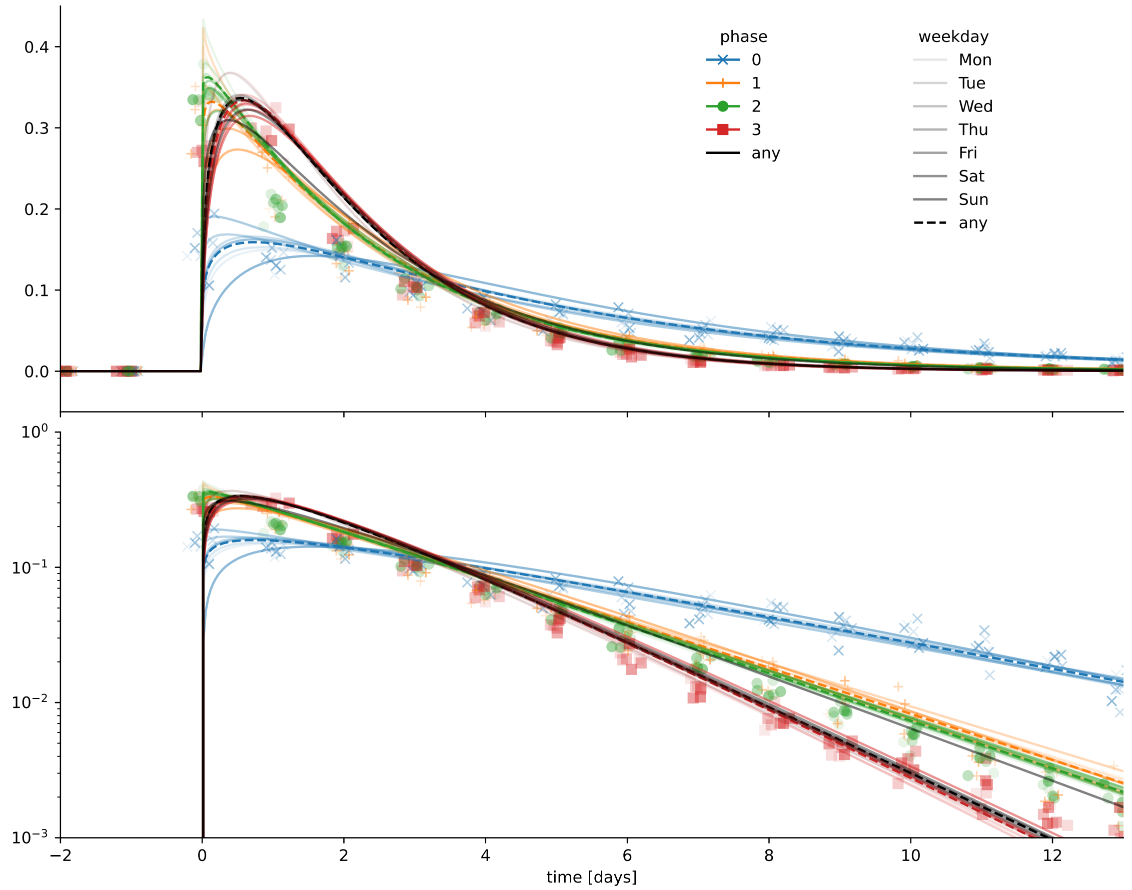

Fig B. Relative frequencies (discrete distributions) of delays provided in the data are shown as markers, the fitted distributions (continuous) are shown as lines. Different colors correspond to different time periods ('phases'), color intensity encodes the day of the week.
