# Supplementary material for: Assessing the heterogeneity in the transmission of infectious diseases from time series of epidemiological data
Source: PLoS One. 2023 May 30;18(5):e0286012. doi: 10.1371/journal.pone.0286012 (PMC10228818; doi:10.1371/journal.pone.0286012)
Supplement: S3 Text — Formal representation of the equations defining the stochastic model for the MCMC inference approach. (PDF) [file pone.0286012.s007.pdf]

S3 Text: Equations and constraints for stochastic simulation

To map the equation system developed in *Results* in the main text in a stochastic simulation framework (S1 Code), we differentiate between distinct instances of random variables and introduce the following intermediate definitions (deterministic combinations of 'exogenous' random variables),

$$\begin{aligned}
\Delta^{1}_{\text{ser}} &:= \Delta^{AB}_{\text{gen}} - \Delta^{A}_{\text{inc}} + \Delta^{B}_{\text{inc}} \\
\Delta^{1}_{\text{case}} &:= \Delta^{AB}_{\text{gen}} - \Delta^{A}_{\text{inc}} - \Delta^{A}_{\text{reg}} + \Delta^{B}_{\text{inc}} + \Delta^{B}_{\text{reg}} \\
\Delta^{2}_{\text{case}} &:= \Delta^{AB}_{\text{ser}} - \Delta^{A}_{\text{reg}} + \Delta^{B}_{\text{reg}} \\
\Delta^{1}_{\text{rep}^*} &:= \Delta^{AB}_{\text{gen}} - \Delta^{A}_{\text{inc}} - \Delta^{A}_{\text{reg}} \\
\Delta^{2}_{\text{rep}^*} &:= \Delta^{AB}_{\text{ser}} - \Delta^{B}_{\text{inc}} - \Delta^{A}_{\text{reg}} \\
\Delta^{A}_{\text{rep}^\dagger} &:= \Delta^{A}_{\text{inc}} + \Delta^{A}_{\text{reg}} \\
\Delta^{B}_{\text{rep}^\dagger} &:= \Delta^{B}_{\text{inc}} + \Delta^{B}_{\text{reg}}.
\end{aligned} \tag{i}$$

We regard the difference between instances of the same variable as stochastic errors ε that are modelled as normal random variables with a standard deviation of 3 days,

$$\begin{aligned}
\varepsilon_{\text{ser}} &:= \Delta^{AB}_{\text{ser}} - \Delta^{1}_{\text{ser}} \\
\varepsilon_{\text{case}} &:= \Delta^{1}_{\text{case}} - \Delta^{2}_{\text{case}} \\
\varepsilon_{\text{rep}^*} &:= \Delta^{1}_{\text{rep}^*} - \Delta^{2}_{\text{rep}^*}.
\end{aligned} \tag{ii}$$
