# Supplementary material for: Assessing the heterogeneity in the transmission of infectious diseases from time series of epidemiological data
Source: PLoS One. 2023 May 30;18(5):e0286012. doi: 10.1371/journal.pone.0286012 (PMC10228818; doi:10.1371/journal.pone.0286012)
Supplement: S4 Text — Analysis of the posterior distributions obtained by stochastic inference. (PDF) [file pone.0286012.s008.pdf]

S4 Text: Validation of the posterior distributions in the MCMC inference approach

In Fig A the prior and posterior parameters of the distributions that occurred during stochastic inference with the MCMC approach are plotted. The underlying scenario is based on the averaged serial interval distribution with strictly positive duration and the reporting delay that was found for Wednesdays during the whole recorded epidemic in Austria (`pSI_weekday3`). The resulting posterior parameters corresponding to the exogenous statistical models only deviate marginally from the original prior parameters, indicating that the original statistical models are not altered during simulation. The same can be recognized in Fig B, which shows the density functions of the prior and posterior statistical models of all disease intervals. The qualitative properties of the obtained distributions of endogenous variables (reporting offset distributions and case interval) also comply with the heuristic characterization of disease intervals in *Methods* in the main text.

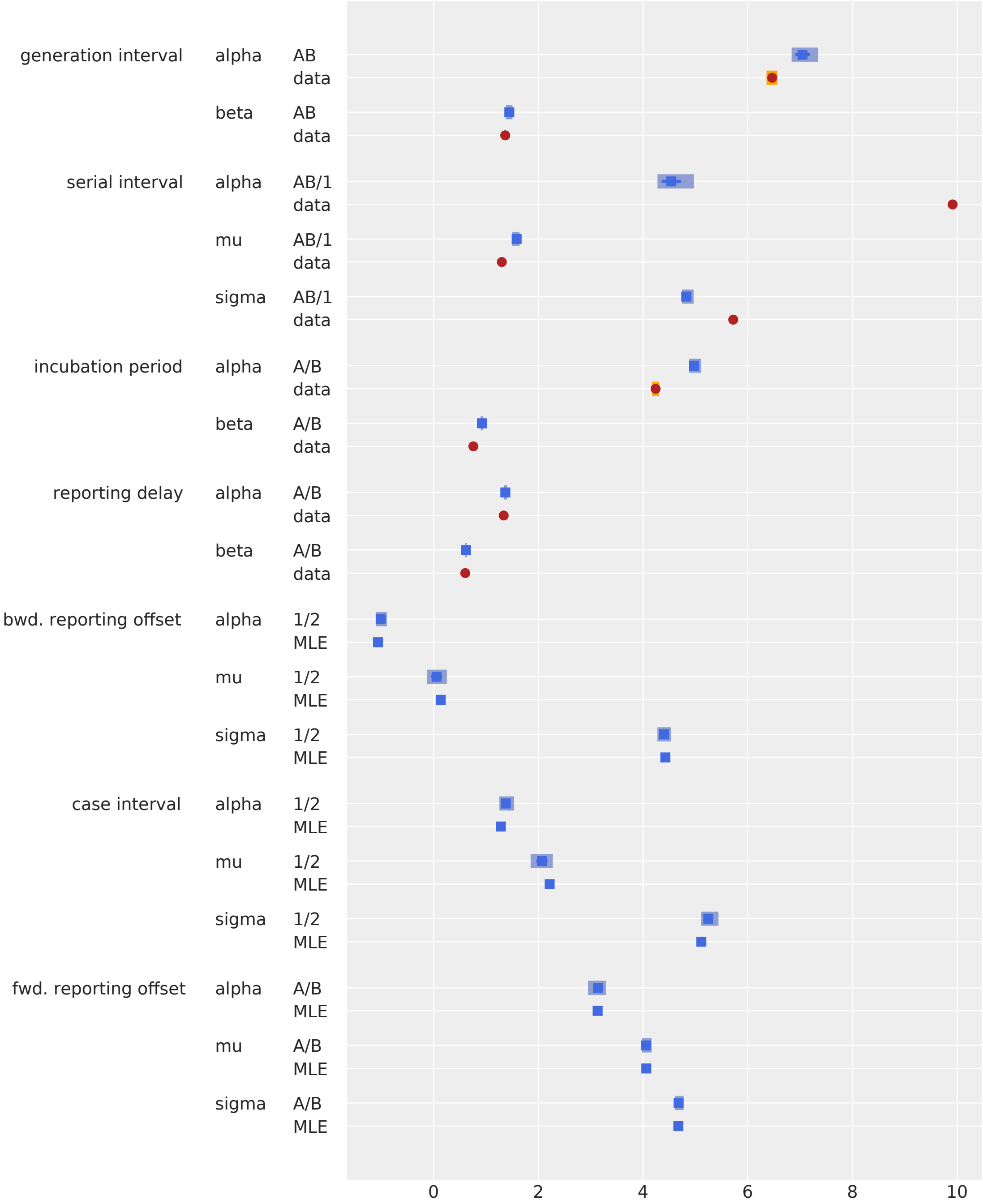

Fig A. Parameters of interval distributions in the stochastic simulation approach. Prior parameters and their credible intervals (if provided by literature and data) are marked as dots in red color and labeled with 'data'. The inferred parameters and their uncertainty intervals are displayed as blue squares. In addition to the parameters obtained via MCMC sampling, we also show the maximum likelihood estimates (indicated by MLE). Visual comparison of the statistical parameters of each disease interval shows that during stochastic simulation the initial models were not altered severely.

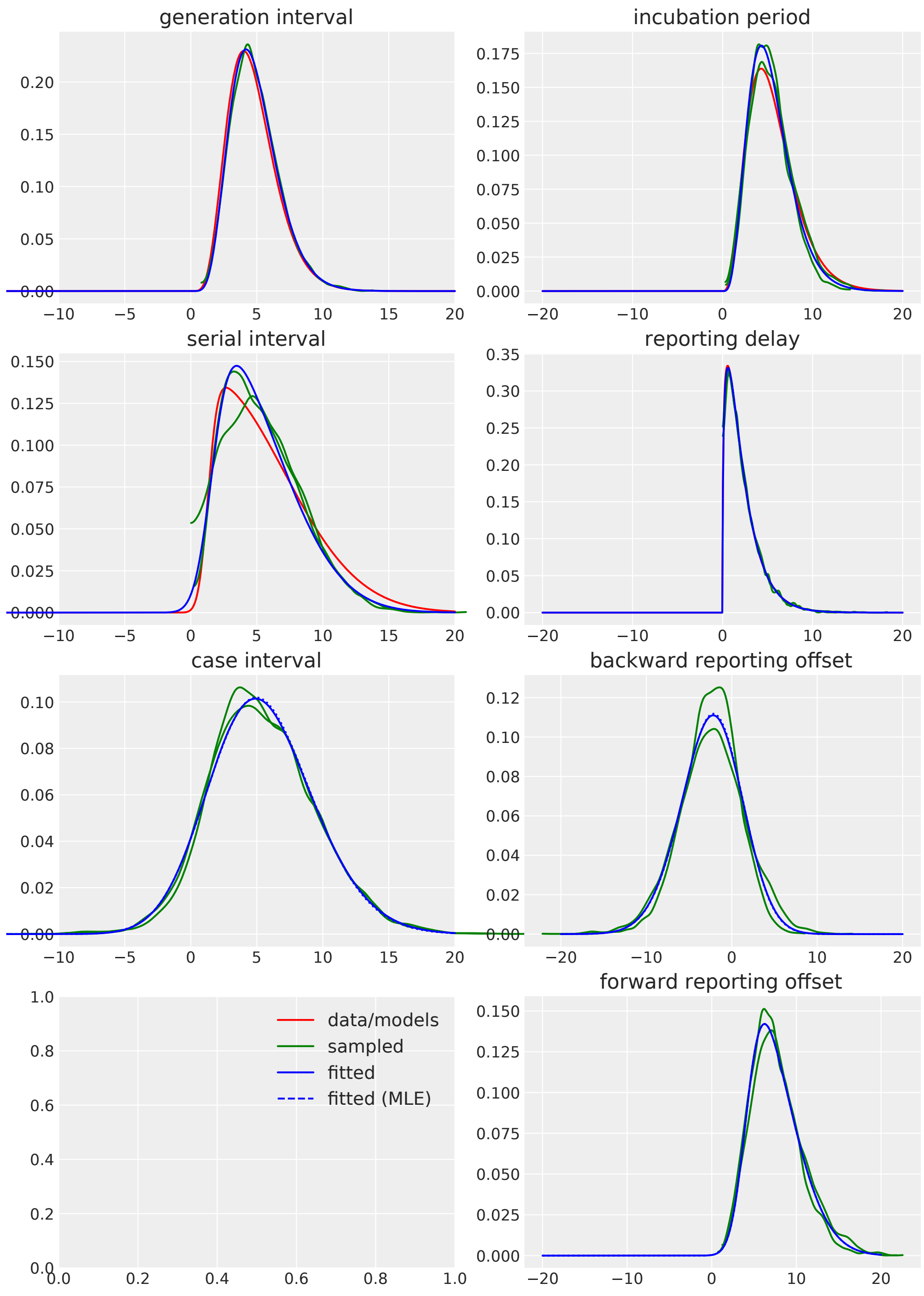

Fig B. Interval distributions in the stochastic simulation approach. A priori interval distributions based on literature and data (red), posterior sample distributions obtained from simulations of the constrained equation model (green); statistical models fitted to the simulation data (blue).
