# Supplementary material for: Assessing the heterogeneity in the transmission of infectious diseases from time series of epidemiological data
Source: PLoS One. 2023 May 30;18(5):e0286012. doi: 10.1371/journal.pone.0286012 (PMC10228818; doi:10.1371/journal.pone.0286012)
Supplement: S5 Text — Visualization of the statistical distributions provided in S3 Data. (PDF) [file pone.0286012.s009.pdf]

S5 Text: Visual display of the inferred disease interval distributions

The inferred reporting offset intervals and the corresponding case interval change significantly over the course of the pandemic, i.e. for different phases of the COVID-19 pandemic in Austria (phase 0: 2020-02 to 2020-05, phase 1: 2020-06 to 2020-09, phase 2: 2020-10 to 2021-02, phase 3: 2021-03 onward). In particular, the variance of the time intervals is larger during the first phase, but settles at a smaller value in the later phases (Fig A). Conversely, the average or mean duration between case reporting and an infection event (reporting offset distributions) is longer during the first phase but gradually declines with time (Fig B). A similar effect at a much smaller scale can be observed for weekends, during which the average reporting intervals are marginally larger.

A possible explanation for the observed changes is the gradual development of testing and reporting procedures over the course of the pandemic, or, respectively, higher availability of testing during business days. Also the emergence of new variants (Alpha became the dominant SARS-CoV-2 variant in Austria in early 2021) with increased infectivity, reduced incubation time, or timely symptom onset, could be a reason for shorter offset intervals.

The observed characteristics are a direct consequence of the measured reporting delays (S2 Text), which were used as exogenous variables in the stochastic inference approach (see S3 Text and S4 Text). Figs A and B are visualizations of the inferred probability distributions describing the reporting offset intervals and the case interval. The corresponding statistical parameters are provided in S3 Data.

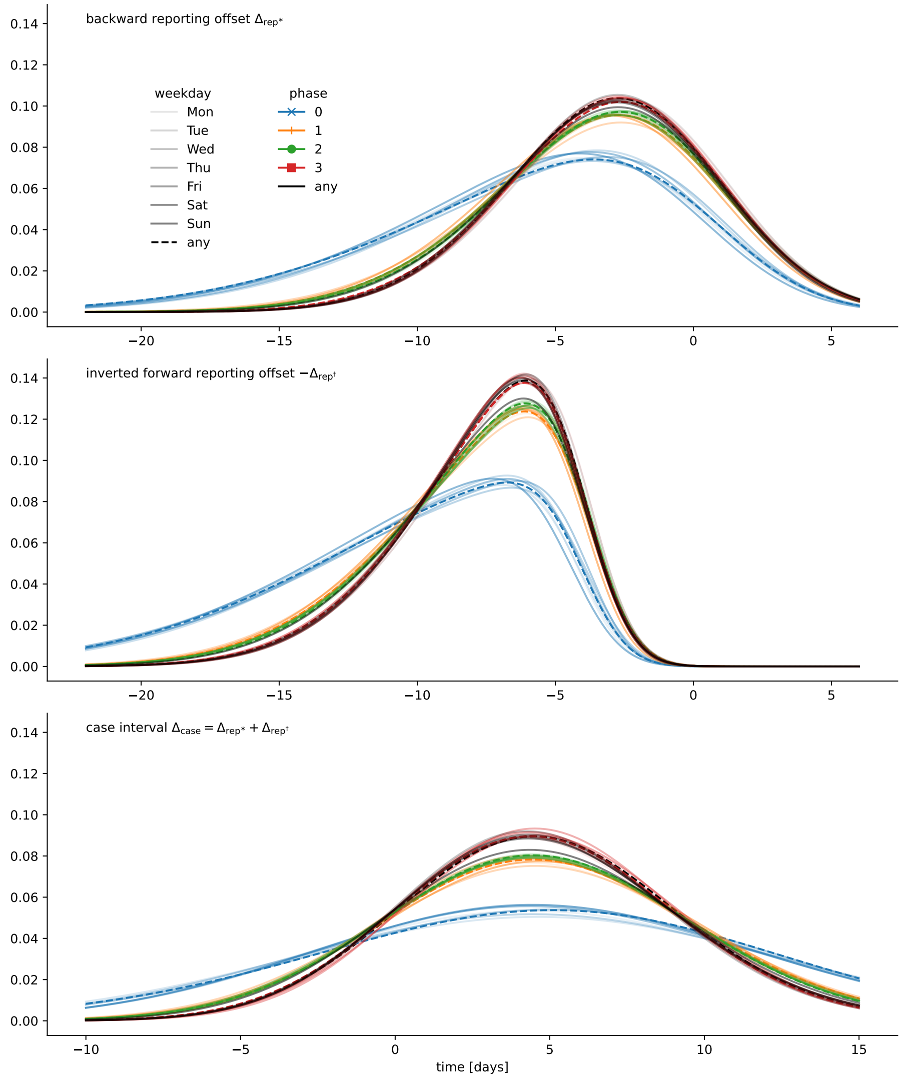

Fig A. Reporting offset distributions obtained for different days of the week and for different phases of the epidemic in Austria. The displayed distributions were obtained by stochastic inference based on the serial interval model that also allows for negative duration ('nSI'). On average, without distinguishing different phases or weekdays (black dashed line), we obtain probability distributions with the mean values and standard deviations $\mu_{\text{rep}^\dagger} = 7.71$, $\mu_{\text{rep}^*} = -3.05$, $\mu_{\text{case}} = 4.69$ and $\sigma_{\text{rep}^\dagger} = 3.15$, $\sigma_{\text{rep}^*} = 3.88$, $\sigma_{\text{case}} = 4.48$.

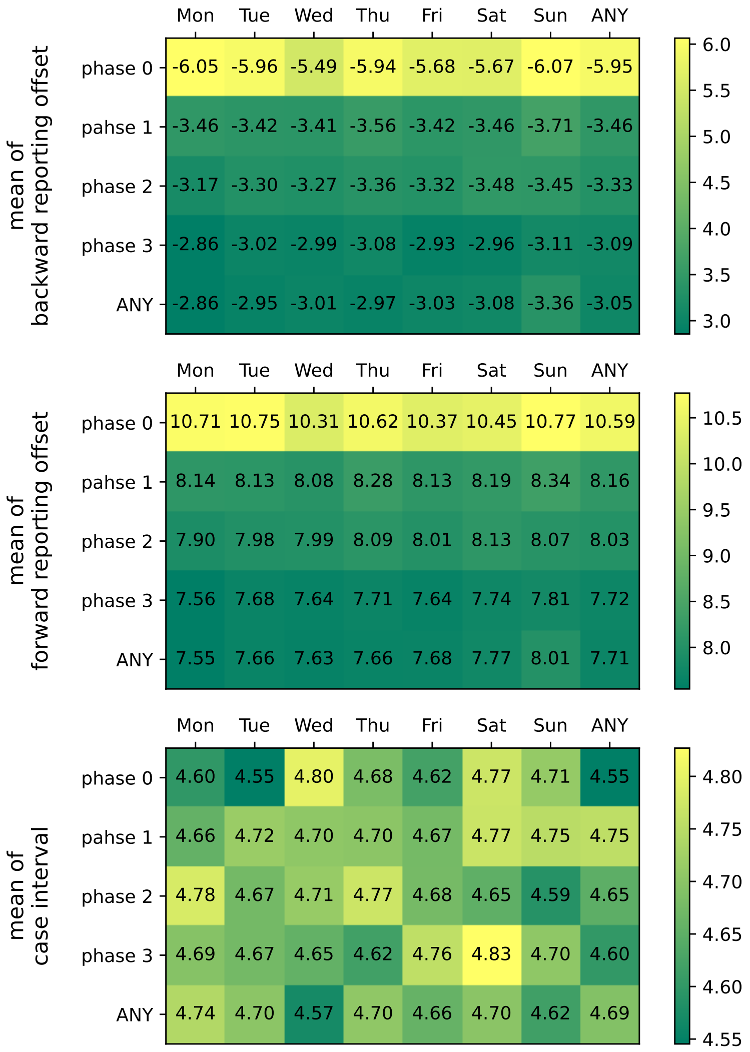

Fig B. Tabular display of the mean values of inferred reporting offset distributions and of the inferred case interval distributions. The used inference model assumes that the serial interval can take negative duration ('nSI').
