# Supplementary material for: Assessing the heterogeneity in the transmission of infectious diseases from time series of epidemiological data
Source: PLoS One. 2023 May 30;18(5):e0286012. doi: 10.1371/journal.pone.0286012 (PMC10228818; doi:10.1371/journal.pone.0286012)
Supplement: S6 Text — Additional visualization of the statistical distributions provided in S3 Data. (PDF) [file pone.0286012.s010.pdf]

S6 Text: Comparison of inferred interval models under different conditions for the positivity of the serial intervals

The figure below provides a visual comparison of the data provided in S3 Data for different assumptions about the serial interval distribution. The serial interval can be modeled with distributions on the whole real line (‘nSI’) and with distributions on the positive line (‘pSI’). Despite appearing small, the differences can become relevant during stochastic simulation.

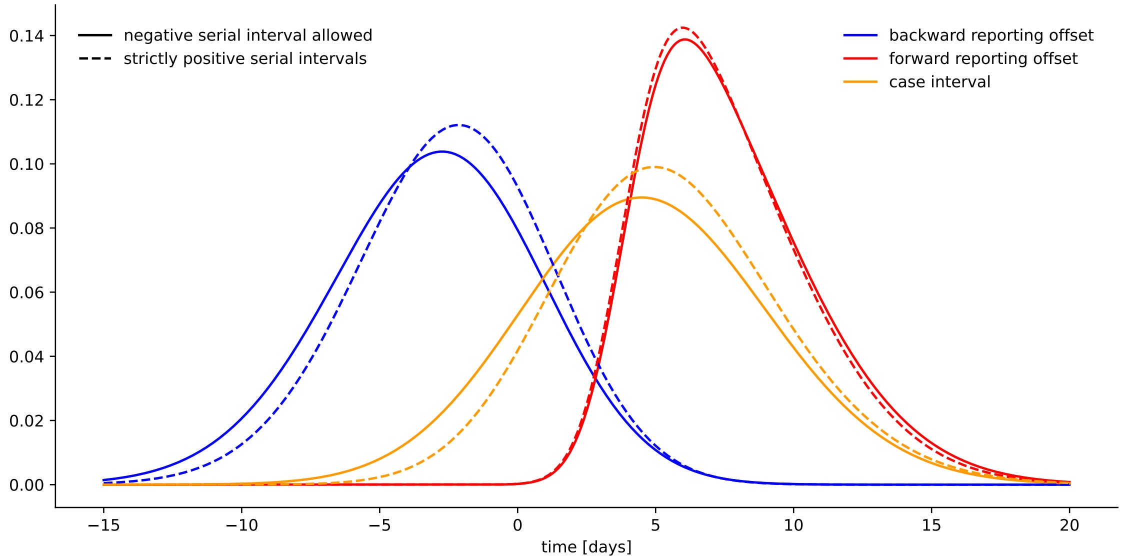

Fig A. Comparison of inferred interval distributions under different conditions for the serial interval distribution (‘nSI’ and ‘pSI’).
