# Supplementary material for: Assessing the heterogeneity in the transmission of infectious diseases from time series of epidemiological data
Source: PLoS One. 2023 May 30;18(5):e0286012. doi: 10.1371/journal.pone.0286012 (PMC10228818; doi:10.1371/journal.pone.0286012)
Supplement: S7 Text — Details on the SSEs that are indicated in Fig 5. (PDF) [file pone.0286012.s011.pdf]

S7 Text: List of selected superspreading events in Austria

A selection of superspreading events in Austria that were reported in the news media. This list is exemplary and only serves to substantiate the quantification of socio-demographic heterogeneity presented in Fig 5 in the main text.

C1 Geographically localized cluster in the state of Tyrol (region code 7), commonly known as the Ischgl incident. Date: 2020-03 References: https://www.politico.eu/article/the-austrian-ski-town-that-spread-coronavirus-across-the-continent/, https://web.archive.org/web/20200323121016/https://www.politico.eu/article/the-austrian-ski-town-that-spread-coronavirus-across-the-continent/

C2 Reports about infection clusters in retirement homes and resulting from social conventions in Vienna and Upper Austria. Date: 2020-03-22 – 2020-03-25 References: https://ooe.orf.at/stories/3040384/, https://kurier.at/chronik/wien/coronavirus-ausbruch-in-vier-wiener-pensionistenheimen/400792283

C3 Multiple larger SSEs (60 infected) in logistic centers and in social amenities in and around Vienna (region code 9). Date: 2020-05-04 – 2020-05-18 References: https://wien.orf.at/stories/3049245/

C4 Multiple larger SSEs in the state of Lower Austria (region code 3). 26 cases in religious event and 40+ cases ins meat factory. Date: 2020-07-17 References: https://noe.orf.at/stories/3058053/

C5 Localized cluster of young and middle-aged persons in tourist destination in St. Wolfgang, Upper Austria (region code 4). 62 infected directly attributed to SSE, 110 secondary infections estimated. Date: 2020-07-27 References: https://www.derstandard.de/story/2000119006698/st-wolfgang-nimmt-den-cluster-gelassen

C6 Reports about infection clusters during private meetings and celebration. Date: 2020-08-14 – 2020-08-18 References: https://www.diepresse.com/5852823/282-neuinfektionen-in-osterreich-die-alterspyramide-hat-sich-vollig-verandert, https://www.sn.at/salzburg/chronik/der-naechste-cluster-in-salzburg-16-covidfaelle-in-einrichtung-der-lebenshilfe-91666543

C7 SSEs (20 to 50 detected cases) in public events and entertainment across the country. Date: 2020-09-14 – 2020-09-15 References: https://www.vienna.at/coronavirus-cluster-an-muk-privatuni-in-wien-auf-46-infizierte-erhoeht/6740633, https://tirol.orf.at/stories/3067024/

C8 Reports about infection clusters (50+ infected) in retirement homes. Date: 2021-01-11 – 2021-01-14 References: https://kurier.at/chronik/wien/virus-mutation-kam-wahrscheinlich-mit-einem-mitarbeiter/401155377 https://salzburg.orf.at/stories/3084397/
