# Supplementary material for: Assessing the heterogeneity in the transmission of infectious diseases from time series of epidemiological data
Source: PLoS One. 2023 May 30;18(5):e0286012. doi: 10.1371/journal.pone.0286012 (PMC10228818; doi:10.1371/journal.pone.0286012)
Supplement: S8 Text — Formal analysis of the reproduction models in Eqs 4–6. (PDF) [file pone.0286012.s012.pdf]

S8 Text: Additional details on the statistical models for reproduction

This is to provide some additional details on the statistical models for reproduction in Eq (4), Eq (5) and Eq (6) in the main text. We use the following parameterizations of probability distributions,

$$\begin{aligned}
\text{NB}(\text{number of failure until stop } r, \text{probability of success } p), \quad & P(x) = \binom{x+r-1}{r-1}(1-p)^r p^x \\
\text{Poisson}(\text{rate } \lambda), \quad & P(x) = \frac{\lambda^x e^{-\lambda}}{x!} \\
\text{Gamma}(\text{shape } s, \text{scale } \theta), \quad & f(x) = \frac{1}{\Gamma(s)\theta^s} x^{s-1} e^{-\frac{x}{\theta}}.
\end{aligned}$$

The mean and the variance of the negative binomial distribution and the gamma distribution agree for the parameter choices

$$\text{NB}(r, p) \qquad \text{and} \qquad \text{Gamma}\left(rp, \frac{1}{1-p}\right).$$

Let X_t bet the expected number of secondary cases produced by the infectious population I_t^*. A common approach for a statistical model for infectious activity is

$$I_t^\dagger \sim \text{Poisson}(X_t). \tag{i}$$

X_t is often modelled as the sum of individual gamma distributed reproduction factors R_i such that

$$I_t^\dagger \sim \text{Poisson}\left(\sum_{i=1}^{I_t^*} R_i\right), \qquad R_i \sim \text{Gamma}(k, R_t/k), \tag{ii}$$

which, as a gamma-Poisson mixture, can be written as (Eq (5) in the main text),

$$I_t^\dagger \sim \text{NB}\left(k\, I_t^*, \frac{R_t}{R_t + k}\right), \tag{iii}$$

where by the summation property of the gamma distribution

$$X_t = \sum_{i=1}^{I_t^*} R_i \sim \text{Gamma}(I_t^* k, R_t/k). \tag{iv}$$

Rewriting

$$X_t = I_t^* \left(\frac{1}{I_t^*} \sum_{i=1}^{I_t^*} R_i\right) \tag{v}$$

and replacing the average individual reproduction factor with $\bar{R}_t \sim \text{Gamma}(k, R_t/k)$, we obtain by the scaling property of the gamma distribution,

$$X_t = I_t^* \bar{R}_t \sim \text{Gamma}(k, I_t^* R_t/k). \tag{vi}$$

The resulting gamma-Poisson mixture for infectious activity is

$$I_t^\dagger \sim \text{NB}\left(k, \frac{I_t^* R_t}{I_t^* R_t + k}\right). \tag{vii}$$

For a broad range of configurations this model can be approximated by

$$I_t^\dagger \sim \mathrm{NB}\left(\frac{I_t^* R_t k}{I_t^* R_t - k}, \frac{I_t^* R_t - k}{I_t^* R_t}\right), \tag{viii}$$

which, in turn, can be approximated by (Eq (6) in the main text)

$$I_t^\dagger \sim \mathrm{Gamma}\left(k, \frac{I_t^* R_t}{k}\right). \tag{ix}$$

The resulting gamma model has a comparably simple parameterization and statistical properties and satisfies the considerations in the paper. In Table A we compare the parameters and main statistical properties of the models discussed above.

model	mean	variance	coefficient of dispersion	coefficient of variation
$I_t^\dagger \sim \mathrm{NB}\left(k\, I_t^*, \frac{R_t}{R_t+k}\right)$<br>$I_t^\dagger \sim \mathrm{Gamma}\left(\frac{k I_t^* R_t}{R_t+k}, \frac{R_t+k}{k}\right)$	$I_t^* R_t$	$k^{-1} I_t^* R_t^2 + I_t^* R_t$	$k^{-1} R_t + 1$	$k^{-1/2}\sqrt{\frac{R_t+k}{I_t^* R_t}}$
$I_t^\dagger \sim \mathrm{NB}\left(k, \frac{I_t^* R_t}{I_t^* R_t+k}\right)$<br>$I_t^\dagger \sim \mathrm{Gamma}\left(\frac{k I_t^* R_t}{I_t^* R_t+k}, \frac{I_t^* R_t+k}{k}\right)$	$I_t^* R_t$	$k^{-1}(I_t^* R_t)^2 + I_t^* R_t$	$k^{-1} I_t^* R_t + 1$	$k^{-1/2}\sqrt{\frac{I_t^* R_t+k}{I_t^* R_t}}$
$I_t^\dagger \sim \mathrm{NB}\left(\frac{I_t^* R_t k}{I_t^* R_t-k}, \frac{I_t^* R_t-k}{I_t^* R_t}\right)$<br>$I_t^\dagger \sim \mathrm{Gamma}\left(k, \frac{I_t^* R_t}{k}\right)$	$I_t^* R_t$	$k^{-1}(I_t^* R_t)^2$	$k^{-1} I_t^* R_t$	$k^{-1/2}$

Table A. Comparison of statistical models for reproduction.

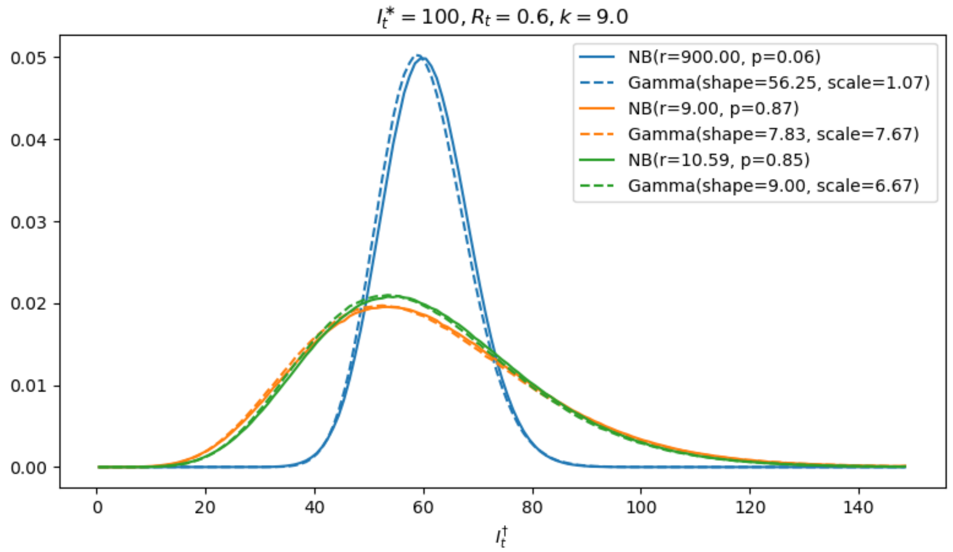

Fig A. Densities of the models in Table A for a choice of parameters. The order in the legend of the figure corresponds to the order in the table.
