# Supplementary material for: Assessing the heterogeneity in the transmission of infectious diseases from time series of epidemiological data
Source: PLoS One. 2023 May 30;18(5):e0286012. doi: 10.1371/journal.pone.0286012 (PMC10228818; doi:10.1371/journal.pone.0286012)
Supplement: S9 Text — EffDI under the variation of the parameters of the statistical distributions of disease intervals. We investigate the robustness of EffDI against the shape of the reporting offset distributions and the behavior of EffDI under the transition between Eqs 2 and 3. (PDF) [file pone.0286012.s013.pdf]

S9 Text: Parameter variation studies

Variation of the mode in the pre-processing of reported case numbers

This supplement investigates the impact of different disease interval models on EffDI. We continuously transform the 'regularizing' model (Eq (2) in the main text) into the 'raw' model (Eq (3) in the main text), which is commonly used for inferring effective reproduction numbers. This is achieved by transforming the parameters (shape, location, scale) of the corresponding disease interval distributions according to

$$\Delta_{\mathrm{rep}^\dagger} \longleftrightarrow \delta_0 \tag{i}$$

$$\Delta_{\mathrm{rep}^*} \longleftrightarrow \Delta_{\mathrm{rep}^*} + \Delta_{\mathrm{rep}^\dagger} = \Delta_{\mathrm{case}} \approx \Delta_{\mathrm{ser}} \approx \Delta_{\mathrm{gen}}. \tag{ii}$$

The transition of the (continuous versions of the) interval distributions is visualized in Fig 1 in the main text and here in Fig A. The parameters γ and δ are used as scaling parameters. On the right-hand side, the delta distribution δ_0 yields the raw reported case numbers as the time series of infectious activity, whereas infectious load is obtained as the convolution with the case interval distribution. Because $\mathbb{E}[\Delta_{\mathrm{rep}^*}] + \mathbb{E}[\Delta_{\mathrm{rep}^\dagger}] = \mathbb{E}[\Delta_{\mathrm{case}}]$, the characteristic inter-case period is retained during the transition.

We see in Fig A that the resulting effective reproduction number is affected only marginally by this transition. However, the obtained EffDI scales towards a homogeneous progression (less distinctive changes in amplitude) with lower detected stochasticity when the 'regularizing' model is approached. This confirms that the regularization of the time series of load and activity impedes the assessment of stochasticity and effective aggregate dispersion in reproduction dynamics.

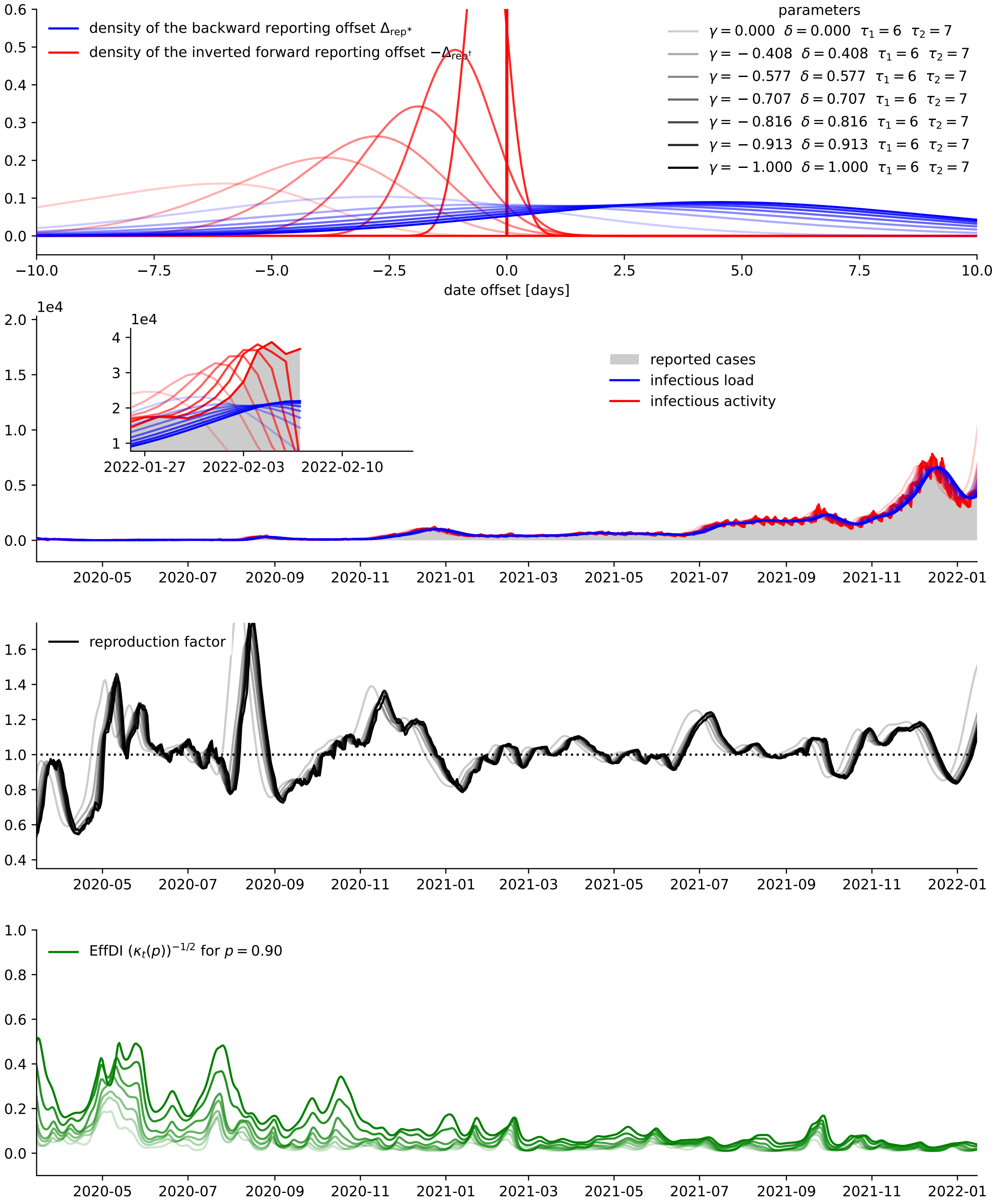

Fig A. Transition between the 'raw' model for load and activity and the 'regularizing' model (Eq (2) and (3) in *Results*). Publicly available data on reported case numbers provided by the Johns Hopkins University Center for Systems Science and Engineering (see reference in the main text) for the SARS-CoV-2 pandemic in South Korea was used. To avoid truncation errors the results were clipped.

Variation of the shape of disease interval distributions

We show that the calculation of a reproduction factor and of EffDI is not sensitive to the shape of the used disease interval distributions. In Fig B the shape parameter of the backward reporting

offset distribution was modulated while holding the mean and variance fixed. For different shapes of the reporting offset, the resulting effective reproduction factor and the EffDI only change marginally. Leaving aside the technical background of the convolution operation, this indicates that for our application merely the mean and variance of disease intervals (backward and forward reporting offset, case interval) are relevant for determining infectious load and activity.

We recapitulate that the distance between the expectation values $\mathbb{E}[\Delta_{\mathrm{rep}^*}] - \mathbb{E}[-\Delta_{\mathrm{rep}^\dagger}] = \mathbb{E}[\Delta_{\mathrm{case}}] \approx \mathbb{E}[\Delta_{\mathrm{ser}}] \approx \mathbb{E}[\Delta_{\mathrm{gen}}]$ controls the inter-case period and that the variance (and abruptness) of the convolution kernels controls the regularization of load and activity. Simultaneous translation of both expectation values, however, results in a shift of the obtained effective reproduction factor and EffDI. In the perspective of real-time assessment and for other applications it might be necessary to exactly model certain properties such as the strictly positive range of disease intervals.

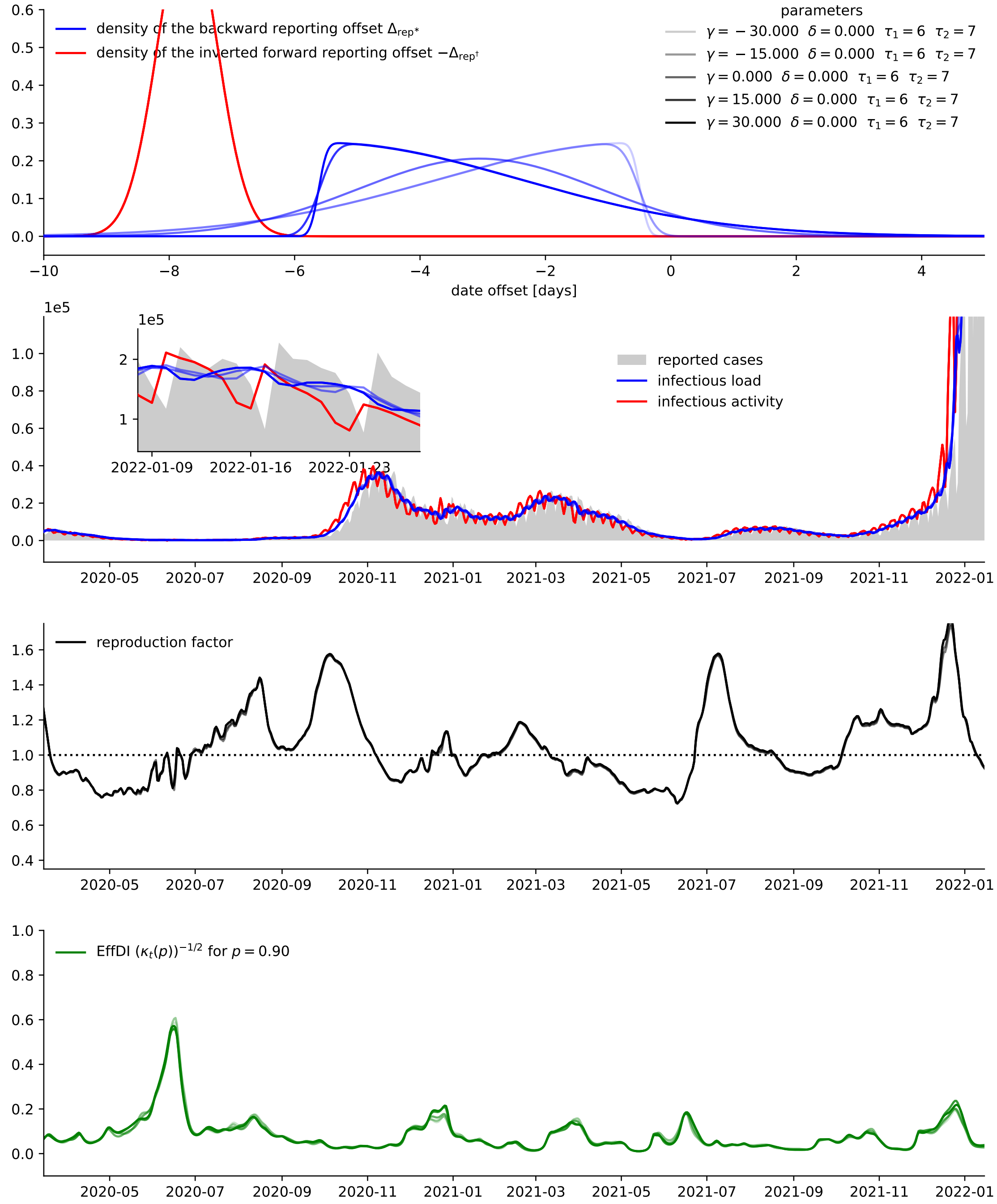

Fig B. Variation of the shape of the backward reporting offset distribution. Publicly available data on reported case numbers provided by the Johns Hopkins University Center for Systems Science and Engineering (see reference in the main text) for the SARS-CoV-2 pandemic in Italy was used. To avoid truncation errors the results were clipped.
