# Supplementary material for: Assessing the heterogeneity in the transmission of infectious diseases from time series of epidemiological data
Source: PLoS One. 2023 May 30;18(5):e0286012. doi: 10.1371/journal.pone.0286012 (PMC10228818; doi:10.1371/journal.pone.0286012)
Supplement: S10 Text — This supporting text compares the emergence of new variants with the behavior of EffDI based on Austrian case data. (PDF) [file pone.0286012.s014.pdf]

S10 Text: Emergence of SARS-CoV-2 variants and EffDI

In the later phase of the SARS-CoV-2 pandemic it became clear that the emergence of new variants can severely impact the spreading dynamics. Especially the Omicron variant featured a distinguished transmissibility when compared to the previous variants. In this supplement we discuss the connection between the behavior of EffDI and the emergence of new variants.

We assume that new variants prevail in the long run if they become dominant by either slowly replacing previous variants or by leading to a new outbreak with significantly increased case numbers. We may even loosely hypothesize that significant peaks in the number of infected are most likely always a result of new variants emerging.

Because the emergence of a new variant often coincides with a change in the infection dynamics we expect a correlation with the behavior of EffDI. In Fig A we recognize in most cases elevated values of EffDI during periods in which a new variant became dominant. However, besides changes in the characteristics of the virus itself, EffDI is also affected by changes in the transmission dynamics that are a result of interventions and administrative processes. At this point it is not possible to draw an exclusive connection between certain patterns in EffDI and the emergence of new variants.

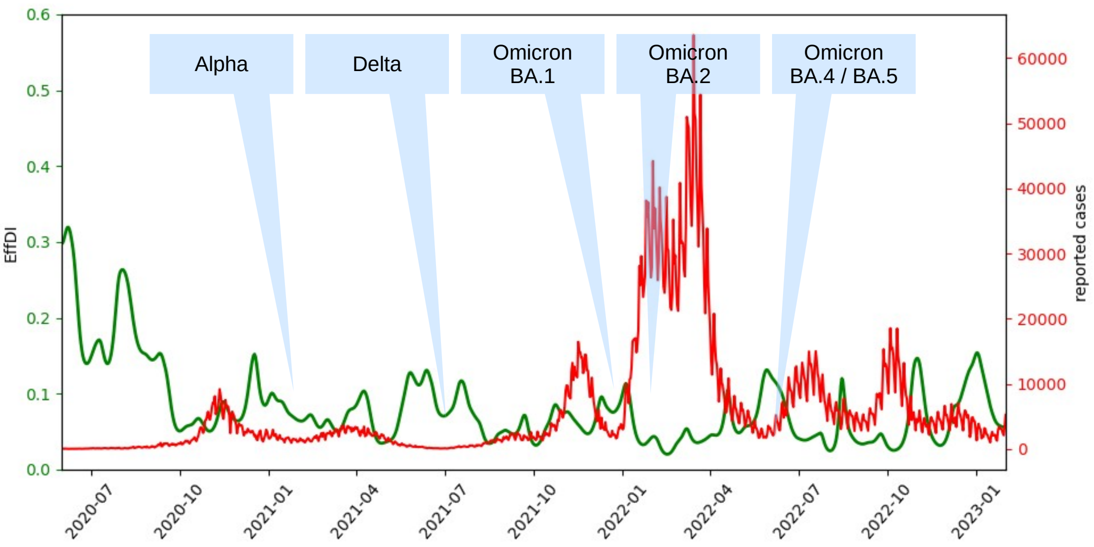

Fig A. Reported case numbers obtained from the official COVID19 Dashboard for Austria (`https://covid19-dashboard.ages.at`) in red color. Derived EffDI in green color. The approximate times when different virus variants became dominant are indicated by blue markers.
